# Diastolic Dysfunction of Aging Is Independent of Myocardial Structure but Associated with Plasma Advanced Glycation End-Product Levels

**DOI:** 10.1371/journal.pone.0049813

**Published:** 2012-11-26

**Authors:** Duncan J. Campbell, Jithendra B. Somaratne, Alicia J. Jenkins, David L. Prior, Michael Yii, James F. Kenny, Andrew E. Newcomb, Casper G. Schalkwijk, Mary Jane Black, Darren J. Kelly

**Affiliations:** 1 St. Vincent’s Institute of Medical Research, Fitzroy, Australia; 2 Department of Medicine, The University of Melbourne, St. Vincent’s Health, Fitzroy, Australia; 3 Department of Cardiology, St. Vincent’s Health, Fitzroy, Australia; 4 Department of Surgery, University of Melbourne, St. Vincent’s Health, Fitzroy, Australia; 5 Department of Cardiothoracic Surgery, St. Vincent’s Health, Fitzroy, Australia; 6 Department of Internal Medicine, University of Maastricht, Maastricht, The Netherlands; 7 Department of Anatomy and Developmental Biology, Monash University, Clayton, Australia; University of Otago, New Zealand

## Abstract

**Background:**

Heart failure is associated with abnormalities of myocardial structure, and plasma levels of the advanced glycation end-product (AGE) N^ε^-(carboxymethyl)lysine (CML) correlate with the severity and prognosis of heart failure. Aging is associated with diastolic dysfunction and increased risk of heart failure, and we investigated the hypothesis that diastolic dysfunction of aging humans is associated with altered myocardial structure and plasma AGE levels.

**Methods:**

We performed histological analysis of non-ischemic left ventricular myocardial biopsies and measured plasma levels of the AGEs CML and low molecular weight fluorophores (LMWFs) in 26 men undergoing coronary artery bypass graft surgery who had transthoracic echocardiography before surgery. None had previous cardiac surgery, myocardial infarction, atrial fibrillation, or heart failure.

**Results:**

The patients were aged 43–78 years and increasing age was associated with echocardiographic indices of diastolic dysfunction, with higher mitral Doppler flow velocity A wave (r = 0.50, *P* = 0.02), lower mitral E/A wave ratio (r = 0.64, *P* = 0.001), longer mitral valve deceleration time (r = 0.42, *P* = 0.03) and lower early diastolic peak velocity of the mitral septal annulus, e’ (r = 0.55, *P* = 0.008). However, neither mitral E/A ratio nor mitral septal e’ was correlated with myocardial total, interstitial or perivascular fibrosis (picrosirius red), immunostaining for collagens I and III, CML, and receptor for AGEs (RAGE), cardiomyocyte width, capillary length density, diffusion radius or arteriolar dimensions. Plasma AGE levels were not associated with age. However, plasma CML levels were associated with E/A ratio (r = 0.44, *P* = 0.04) and e’ (r = 0.51, *P* = 0.02) and LMWF levels were associated with E/A ratio (r = 0.49, *P* = 0.02). Moreover, the mitral E/A ratio remained correlated with plasma LMWF levels in all patients (*P* = 0.04) and the mitral septal e’ remained correlated with plasma CML levels in non-diabetic patients (*P* = 0.007) when age was a covariate.

**Conclusions:**

Diastolic dysfunction of aging was independent of myocardial structure but was associated with plasma AGE levels.

## Introduction

Aging is accompanied by increased vascular and ventricular stiffness, diastolic dysfunction and an increased risk of heart failure [1]–[4]. Heart failure, with either reduced or preserved ejection fraction, is associated with abnormalities of myocardial structure and microvasculature including increased fibrosis, cardiomyocyte hypertrophy and reduced microvascular density [5]–[9], and animal models suggest that these abnormalities precede the development of heart failure in older age. Senescent animals have reduced cardiomyocyte number, hypertrophy of surviving cardiomyocytes, increased cardiac fibrosis, reduced capillary density and increased diffusion radius [10]–[13]. In addition, advanced glycation end-products (AGEs) are proposed to contribute to the increased myocardial stiffening of aging by cross-linking collagen and elastin and promoting collagen accumulation [14], and by promoting inflammation and oxidative stress mediated by the receptor for AGEs (RAGE) [15]. Moreover, plasma AGE levels correlate with the severity and prognosis of heart failure and predict all-cause and cardiovascular disease mortality in older adults [16], [17]. There is, however, only limited information about the effects of aging on the structure and microvasculature of the human myocardium, which comes mainly from autopsy studies that may have been influenced by comorbidities [18]–[20].

We investigated the hypothesis that diastolic dysfunction of aging humans is associated with altered myocardial structure and plasma AGE levels. We performed histological analysis of non-ischemic left ventricular (LV) myocardial biopsies from patients without heart failure or previous myocardial infarction who were undergoing coronary artery bypass graft surgery and had transthoracic echocardiography before surgery. We measured total, interstitial and perivascular myocardial fibrosis, cardiomyocyte size, capillary length density, diffusion radius and arteriolar dimensions. We also measured myocardial expression of the AGE N^ε^-(carboxymethyl)lysine (CML) and RAGE, and plasma levels of CML, low molecular weight fluorophores (LMWFs) and soluble RAGE. Although we obtained LV biopsies from both men and women, preliminary analysis showed gender-specific differences in myocardial structure [21]; therefore, given the smaller number of women recruited to this study, the present analysis was confined to men. We previously reported that neither diabetes nor the metabolic syndrome was associated with altered myocardial total or interstitial fibrosis, cardiomyocyte width, capillary length density, diffusion radius, arteriolar dimensions or immunostaining for collagens I and III, CML, or RAGE in this patient population, although diabetic and metabolic syndrome patients had reduced perivascular fibrosis [22].

## Methods

The St. Vincent’s Health Human Research Ethics Committee (Fitzroy, Australia) approved the study protocol. Each participant provided written informed consent to be included in the study.

### Patients

Details of the Cardiac Tissue Bank have been previously described [21]–[23]. From the Tissue Bank we selected all of 26 male patients having coronary artery bypass graft surgery alone who had transthoracic echocardiography before surgery; none had heart failure or atrial fibrillation, had received loop diuretic therapy or had evidence of previous myocardial infarction. Absence of previous myocardial infarction was established from the clinical history, electrocardiogram and troponin measurements, and was confirmed by inspection of the ventriculogram, transthoracic and transesophageal echocardiography and examination of the heart at surgery. All patients had normal or near-normal LV systolic function as assessed by pre-operative transthoracic echocardiography and ventriculogram, with LV ejection fraction ≥50%. A partial-thickness wedge-shaped biopsy was taken during surgery, immediately after cardioplegia, from a region of the lateral wall of the LV near the base of the heart, between the territories of the left anterior descending and circumflex arteries, that was free of any macroscopic pathology, without evidence of ischemia or wall motion abnormality on pre-operative or intra-operative imaging studies.

The metabolic syndrome was defined according to the International Diabetes Federation [24]. For patients in whom abdominal circumference was not measured, based on the relationship between abdominal circumference and BMI [25], those with BMI >25 kg/m^2^ were considered to exceed the abdominal circumference threshold for the metabolic syndrome. A patient had diabetes if a history of diabetes was evident from use of glucose-lowering medications and/or insulin or if fasting plasma glucose was ≥7 mmol/L [26]. All 6 diabetic patients had type 2 diabetes; one was newly diagnosed and treated with diet alone, two were treated with insulin alone, one with insulin and metformin, and two with metformin and gliclazide. The mean duration of diabetes was 13 (range 0–30) years and the mean HbA1c was 7.5% (range 5.3–9.8%, n = 5).

### Preoperative Transthoracic Echocardiography and Intra-operative Hemodynamics

Preoperative transthoracic echocardiography was performed and reported by either the referring institution or by St. Vincent’s Health according to the American Society of Echocardiography guidelines [27]. Left ventricular mass was calculated using the Devereux equation and left ventricular mass index was calculated by dividing left ventricular mass by height^2.7^ [28], [29]. All patients had Swan-Ganz catheters inserted before surgery that provided measures of pulmonary artery and pulmonary capillary wedge pressures and cardiac output recorded immediately after induction of anesthesia.

### Biochemistry

Blood hemoglobin and hemoglobin A1c and plasma levels of creatinine were measured as part of the routine pre-surgery workup. All other variables were measured on fasting blood collected on the day of surgery, before induction of anesthesia. Estimated glomerular filtration rate (eGFR) was calculated from the Modification of Diet in Renal Disease formula [30]. Insulin resistance (HOMA2-IR), insulin sensitivity (HOMA2-%S) and ß-cell function (HOMA2-%B) were calculated using the HOMA calculator version 2.2 [31]. CML was measured by ELISA (Microcoat, Penzberg, Germany). LMWFs were measured by fluorescence spectroscopy [32]. Soluble RAGE was measured by ELISA (R&D Systems Inc., Minneapolis, MN). Amino-terminal-pro-B-type natriuretic peptide (NT-proBNP) was measured by electrochemiluminescence immunoassay using an Elecsys instrument (Roche Diagnostics, Basel, Switzerland).

### Histological Analysis

Details of tissue collection, fixation and histology have been previously described [21]–[23]. All histological analyses were performed blind to patient identity and age. Picrosirius red-stained 4 µm sections of paraffin-embedded tissue were analyzed for total, interstitial and perivascular fibrosis and arteriolar dimensions by quantitative morphometry of digitized images of the whole myocardial section (Aperio Technologies, Inc., CA) as previously described [21]–[23]. Myocardial total fibrosis was calculated using the positive pixel count algorithm as the area of collagen staining expressed as a percentage of the total myocardial tissue area, after excluding the pericardium, whereas interstitial fibrosis was calculated as described for total fibrosis, with exclusion of perivascular fibrosis.

Arterioles were identified by the presence of a layer of media and immunohistochemical staining for elastin showed the blood vessels were relaxed. The tissue was immersion fixed and the arterioles were usually oval in shape because of deformation and/or because they were cut at an oblique angle. We did not attempt to analyze arterioles in longitudinal section and only arterioles in approximate cross- or oblique-section were analyzed for perivascular fibrosis and arteriolar dimensions; these arterioles had diameters (average of maximum and minimum diameter of each arteriole) of 12–106 µm. Perivascular fibrosis was calculated as the ratio of the area of perivascular fibrosis to the total vessel area (area of vessel wall plus lumen) [33]. Arteriolar wall area/circumference ratio was measured for arterioles with average diameters of 20–80 µm, which represented 88% of all arterioles analyzed.

Cardiomyocyte width, determined on 4 µm sections of paraffin-embedded tissue (one section per patient) stained for reticulin [34], was the mean of >100 measurements for each section of the shortest diameter of cardiomyocyte profiles containing a nucleus.

Capillary length density, which is the length of capillaries per unit volume of tissue, and diffusion radius were determined by analysis of 4 µm sections of paraffin-embedded tissue (one section per patient) immunostained for CD31 (mouse anti-human CD31 monoclonal antibody, Dako Denmark A/S, Glostrup, Denmark) using standard stereological techniques [35]–[38], as previously described [21]–[23].

Immunohistochemistry for collagens I and III was performed in frozen sections using mouse monoclonal antibodies ab6308 and ab6310 (Abcam, Cambridge, UK), respectively. Myocardial total collagen I and III densities were calculated using the positive pixel count algorithm (Aperio Technologies, Inc., CA) as the area of collagen staining expressed as a percentage of the total myocardial tissue area, after excluding the pericardium. Immunohistochemistry for CML was performed in paraffin sections using a mouse monoclonal antibody as described by Schalkwijk et al. [39]. Immunohistochemistry for RAGE was performed with goat polyclonal antibody AB5484 (Millipore, Billerica, MA). Immunostaining of arteriolar media and intima for CML and of arteriolar media, intima and capillaries for RAGE was individually scored by its intensity as 0+, 1+, 2+, or 3+, after inspection of the digitized image of the whole of each section.

### Statistical Analysis

Data are shown as means±SEM or n (%). Correlations were estimated using Pearson correlation coefficients and were considered significant at *P*<0.05.

## Results

### Study Patients

The clinical, biochemical and hemodynamic characteristics of the study patients (age range 43–78 years) are shown in Table 1. Older age was associated with increased plasma NT-proBNP levels (*P* = 0.001) and reduced eGFR (*P* = 0.03). The extent of coronary artery disease (proportions of patients with left main stenosis, three-vessel stenosis, occluded coronary arteries, coronary collaterals and wall motion abnormalities) and number of bypass grafts performed were unrelated to age. In addition, diabetes, metabolic syndrome, medication use and hemodynamics, including pulmonary capillary wedge pressure, were not associated with age.

**Table 1:** Clinical, biochemical and hemodynamic characteristics of men undergoing coronary artery bypass graft surgery who had transthoracic echocardiography before surgery.

Characteristic	Mean±SEM or n (%), n = 26
Mean age (years)	65±2
Left main stenosis >50%, n (%)	14 (54%)
One vessel stenosis >70%, n (%)	4 (15%)
Two vessel stenosis >70%, n (%)	16 (62%)
Three vessel stenosis >70%, n (%)	6 (23%)
Patients with occluded coronary artery, n (%)	10 (38%)
Coronary collaterals, Rentrop grade 2 or 3, n (%)	13 (50%)
Previous percutaneous transluminal coronary angioplasty, n (%)	4 (15%)
Wall motion abnormality, n (%)	3 (12%)
Coronary grafts/patient (n)	3.3±0.2
Body mass index (kg/m^2^)	29.0±0.9
Body surface area (m^2^)	2.01±0.03
Clinical risk factors	
Diabetes, n (%)	6 (23%)
Metabolic syndrome (non-diabetic), n (%)	14 (54%)
Pre-admission systolic blood pressure (mmHg)	134±2
Pre-admission diastolic blood pressure (mmHg)	78±1
Pre-admission pulse pressure (mmHg)	56±2
Previous hypertension, n (%)	17 (65%)
Use of tobacco, ever, n (%)	17 (65%)
Fasting plasma total cholesterol (mmol/L)	3.5±0.2
Fasting plasma LDL cholesterol (mmol/L)	2.0±0.2
Fasting plasma HDL cholesterol (mmol/L)	0.91±0.04
Fasting plasma triglyceride (mmol/L)	1.6±0.2
Fasting plasma glucose (mmol/L)	6.5±0.3
Fasting plasma insulin (pmol/L)	57±11
ß cell function from HOMA2-%B	83±9
Insulin sensitivity from HOMA2-%S	93±13
Insulin resistance from HOMA2-IR	1.6±0.2
Plasma CML (µmol/L)	2.1±0.1
Plasma LMWF (AU/mL)	2.7±0.2
Plasma soluble RAGE (pg/mL)	698±63
Plasma NT-proBNP (pmol/L)	15±2
Hemoglobin (g/L)	14.1±0.3
Plasma creatinine (µmol/L)	95±3
eGFR (mL/min per 1.73 m^2^)	72±3
C-reactive protein (mg/L)	4.7±1.4
Medications	
ACE inhibitor therapy, n (%)	13 (50%)
ARB therapy, n (%)	7 (27%)
ACEI and/or ARB therapy, n (%)	20 (77%)
Statin therapy, n (%)	23 (88%)
Aspirin therapy, n (%)	24 (92%)
Calcium antagonist therapy, n (%)	5 (19%)
ß-blocker therapy, n (%)	16 (62%)
Long-acting nitrate therapy, n (%)	5 (19%)
Thiazide or indapamide therapy, n (%)	2 (8%)
Intra-operative hemodynamics immediately post induction of anesthesia	
Central venous pressure (mmHg)	8.6±0.9
Pulmonary capillary wedge pressure (mmHg)	10.3±0.7
Cardiac index (L/min/m^2^)	2.7±0.2
Stroke Volume index (mL/m^2^)	47±2

Coronary collaterals were scored according to Rentrop et al. [58]. ACE, angiotensin converting enzyme; ARB, angiotensin receptor blocker; eGFR, estimated glomerular filtration rate calculated using the Modification of Diet in Renal Disease study equation [30]; CML, N^ε^-(carboxymethyl)lysine; HDL, high density lipoprotein; HOMA, Homeostasis Model Assessment calculator version 2.2 [31]; LDL, low density lipoprotein; LMWF, low molecular weight fluorophore; NT-proBNP, amino-terminal-pro-B-type natriuretic peptide; RAGE, receptor for advanced glycation end-products.

### Echocardiography

**Figure 1:**
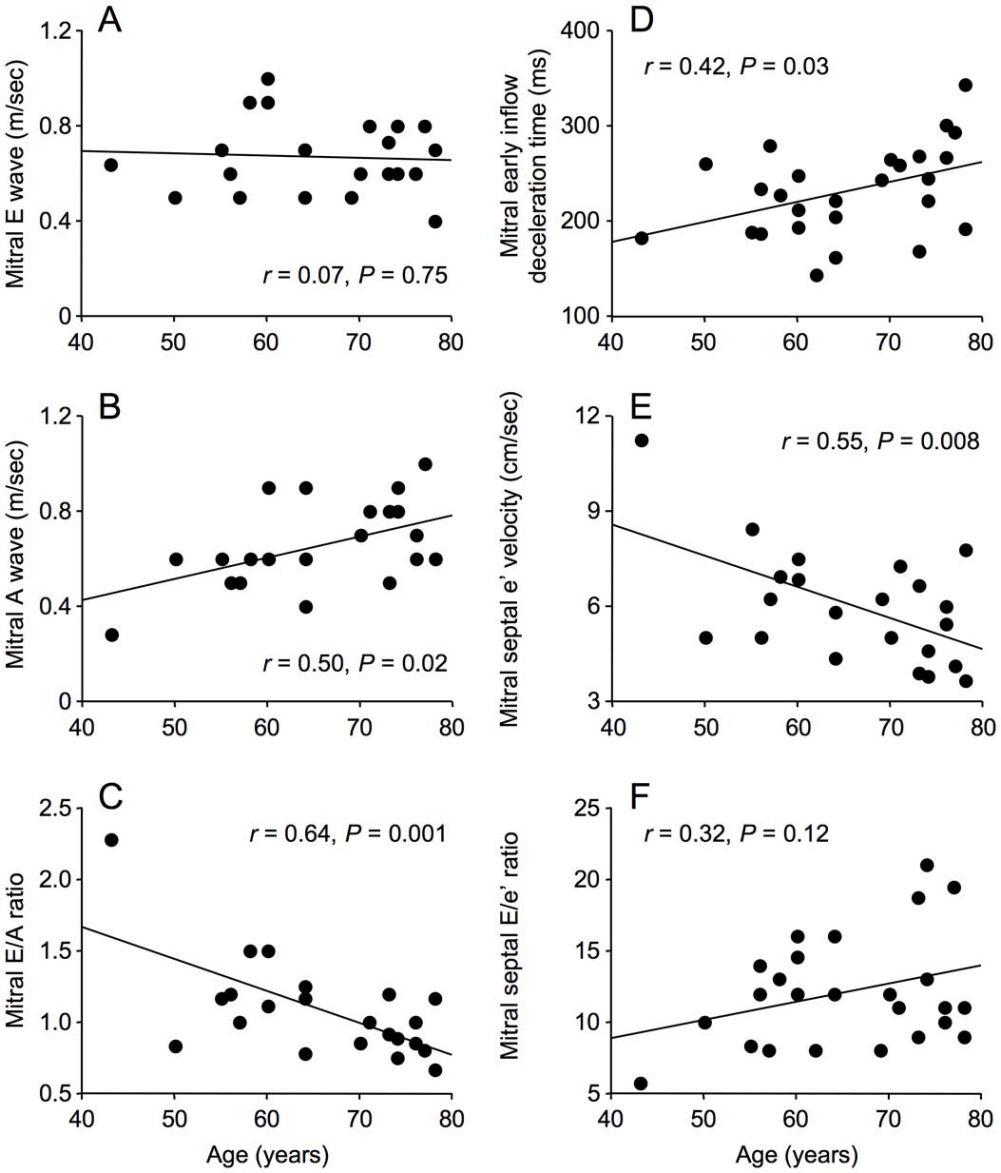
Correlations between echocardiographic parameters and age in men with coronary artery disease. Age was not correlated with mitral Doppler flow velocity E wave velocity (A), but was correlated with mitral Doppler flow velocity A wave velocity (B), E/A ratio (C), mitral early inflow deceleration time (D) and mitral early diastolic peak velocity of the septal mitral annulus, e’ (E). However, the correlation of age with E/e’ ratio was not statistically significant (F).

Age was not associated with LV ejection fraction, left atrial dimensions, LV mass (not shown) or mitral E wave velocity (Figure 1). However, increasing age was associated echocardiographic indices of LV diastolic dysfunction, with higher mitral Doppler flow velocity A wave, lower E/A wave ratio, longer mitral valve deceleration time, and lower early diastolic peak velocity of the mitral septal annulus, e’, although the association between age and E/e’ ratio did not achieve statistical significance (Figure 1). None of the echocardiographic parameters was associated with the presence of wall motion abnormalities or with pulse pressure.

### Histology

Given that the strongest correlations between age and echocardiographic indices of diastolic dysfunction were for mitral E/A ratio and mitral septal e′ velocity, we examined the correlation between these echocardiographic parameters and myocardial histology.

**Figure 2:**
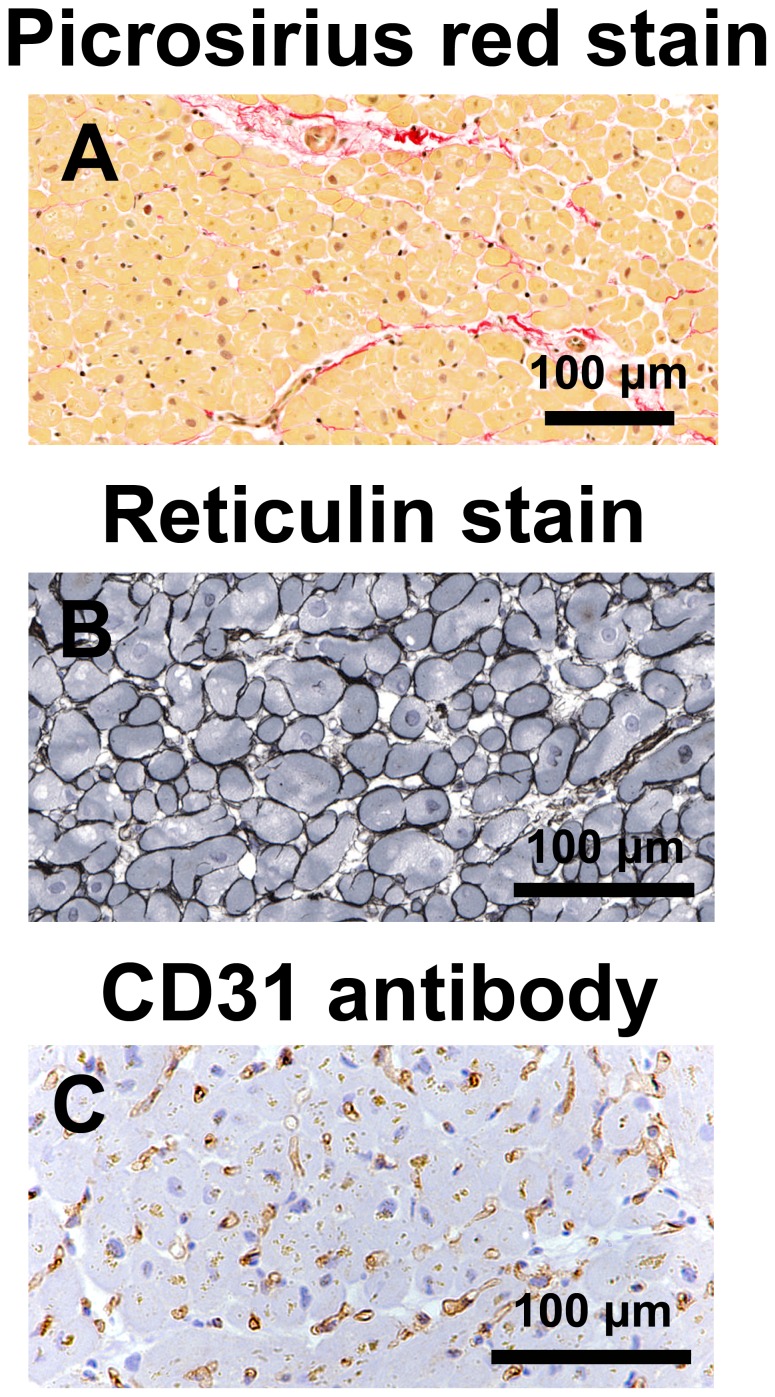
Picrosirius-red staining of collagen, reticulin staining of cardiomyocyte membranes, and CD31 immunostaining of capillaries. Representative sections of left ventricular biopsies from coronary artery bypass graft surgery patients stained with picrosirius-red demonstrating interstitial and perivascular fibrosis (stained red) and arteriolar dimensions (A), reticulin stain demonstrating cardiomyocyte membranes (B), and immunostained for CD31 demonstrating capillaries (C).

**Figure 3:**
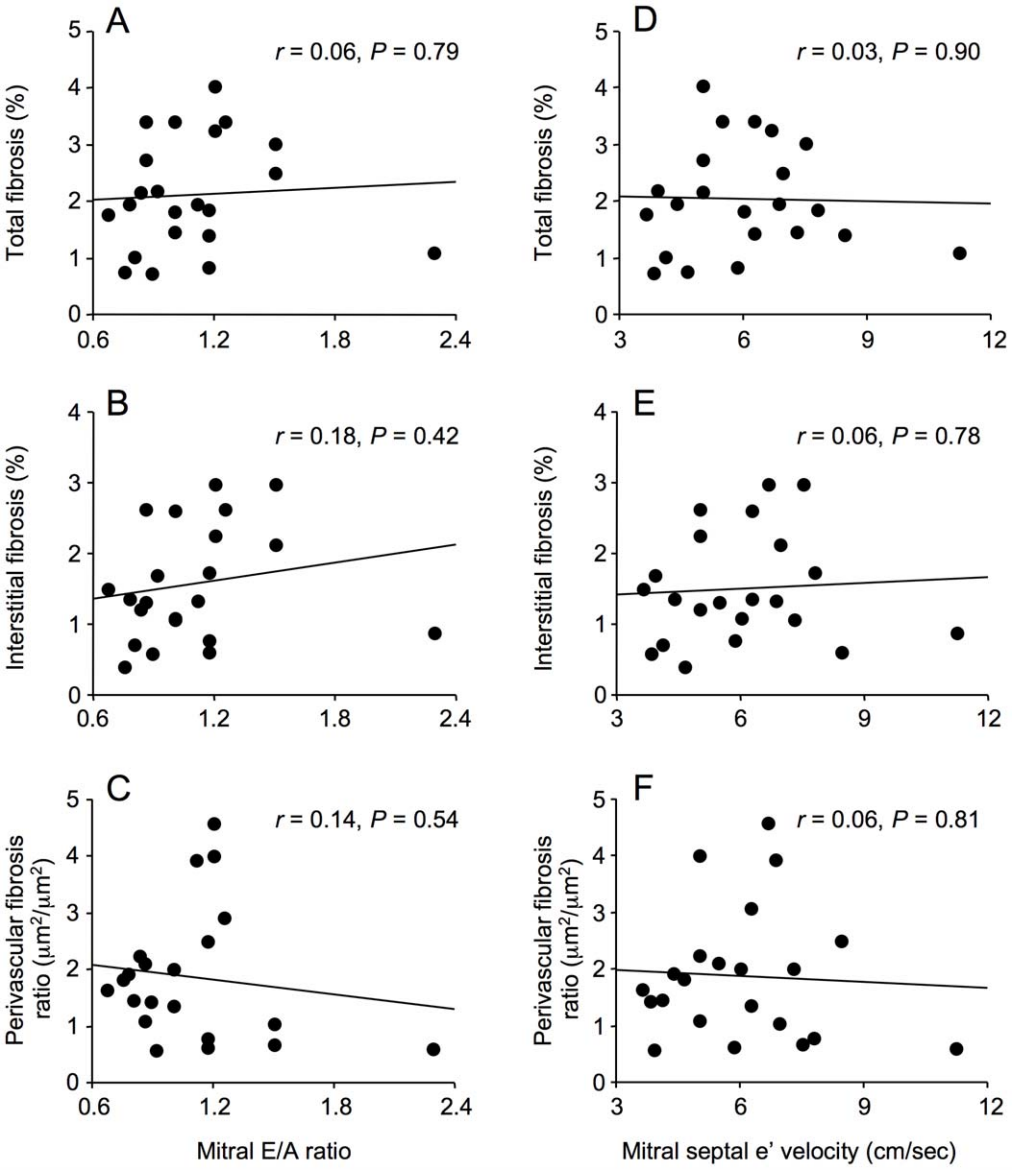
Correlations between myocardial fibrosis and echocardiographic parameters of diastolic dysfunction, mitral E/A ratio and mitral septal e ’ **velocity, in men with coronary artery disease.** Myocardial total fibrosis, interstitial fibrosis and perivascular fibrosis were not correlated with mitral E/A ratio (A, B, C) or with mitral septal e’ velocity (D, E, F).

**Figure 4:**
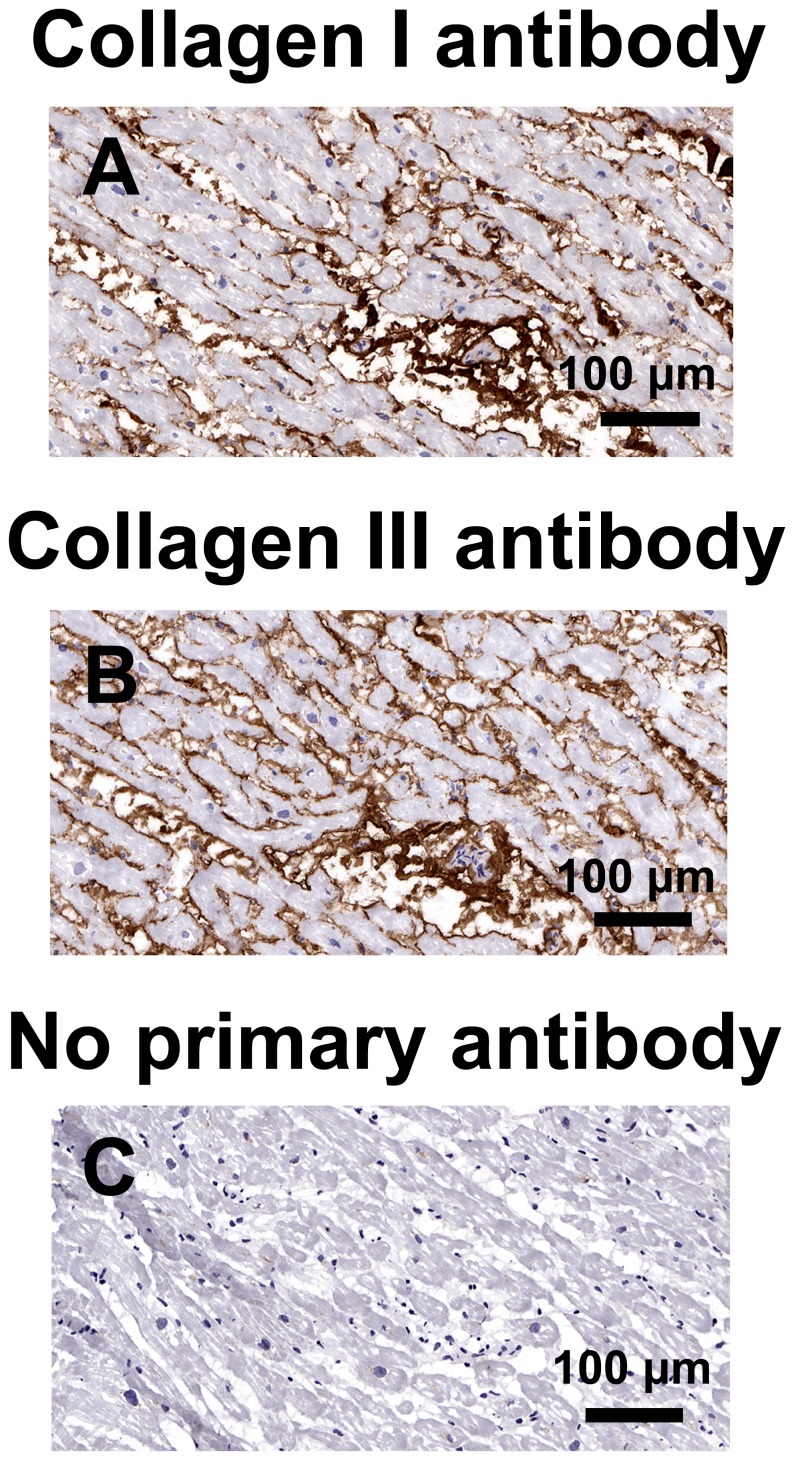
Immunostaining for collagens I and III. Representative sections of a left ventricular biopsy from a coronary artery bypass graft surgery patient immunostained for collagen I (A), collagen III (B), and a negative control section without primary antibody (C).

**Figure 5:**
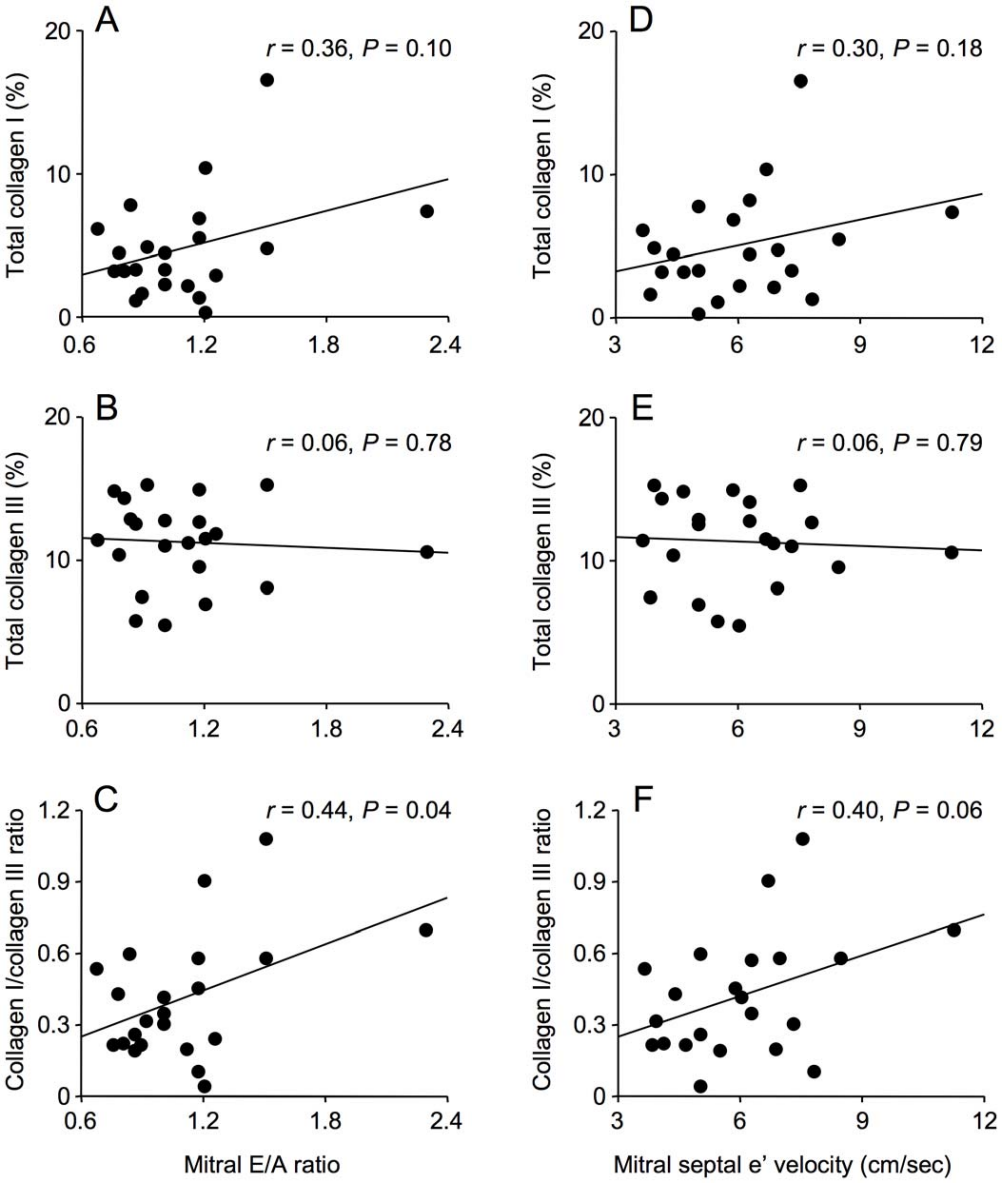
Correlations between collagens I and III as assessed by immunostaining and echocardiographic parameters of diastolic dysfunction, mitral E/A ratio and mitral septal e ’ **velocity, in men with coronary artery disease.** Total collagens I and III were not correlated with mitral E/A ratio (A, B) or with mitral septal e’ velocity (D, E) whereas the correlations between collagen I/collagen III ratio and mitral E/A ratio (C) and mitral septal e’ velocity (F) were of borderline statistical significance.

The area of myocardial paraffin sections (median 4.3 mm^2^, 1.7–10.6 mm^2^) was unrelated to patient age. Myocardial total, interstitial and perivascular fibrosis were not associated with either mitral E/A ratio or mitral septal e’ velocity (Figure 2 and 3). In addition, myocardial total collagen I and III immunostaining were not associated with mitral E/A ratio or mitral septal e’ velocity, although these echocardiographic parameters were weakly associated with collagen I/collagen III ratio (Figure 4 and 5).

**Figure 6:**
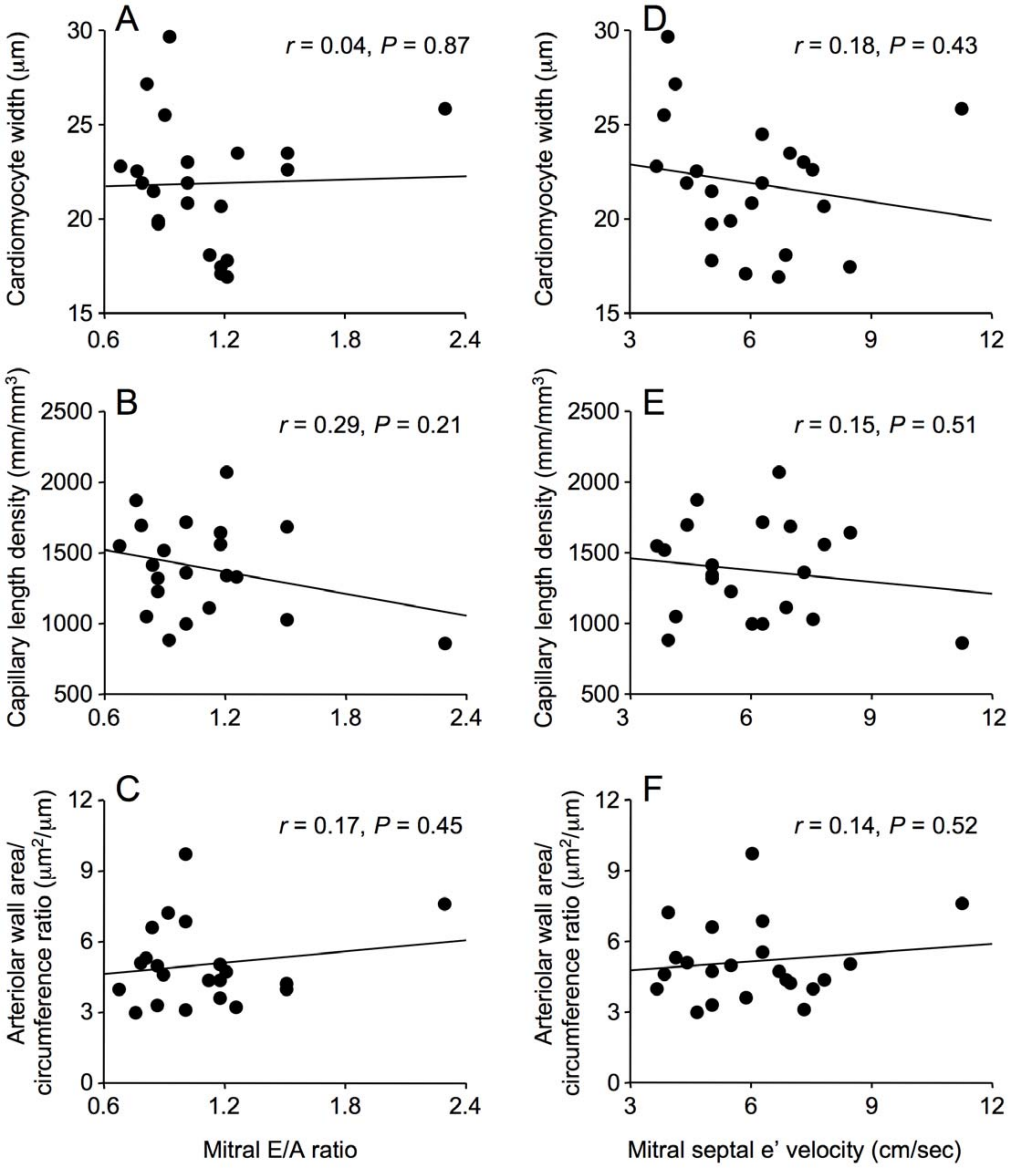
Correlations between cardiomyocyte width, capillary length density and arteriolar wall area/circumference ratio and echocardiographic parameters of diastolic dysfunction, mitral E/A ratio and mitral septal e ’ **velocity, in men with coronary artery disease.** Cardiomyocyte width, capillary length density and arteriolar wall area/circumference ratio were not correlated with mitral E/A ratio (A, B, C) or with mitral septal e’ velocity (D, E, F).

Cardiomyocyte width, capillary length density, diffusion radius (not shown) and arteriolar wall area/circumference ratio were not associated with either mitral E/A ratio or mitral septal e’ velocity (Figure 6).

**Figure 7:**
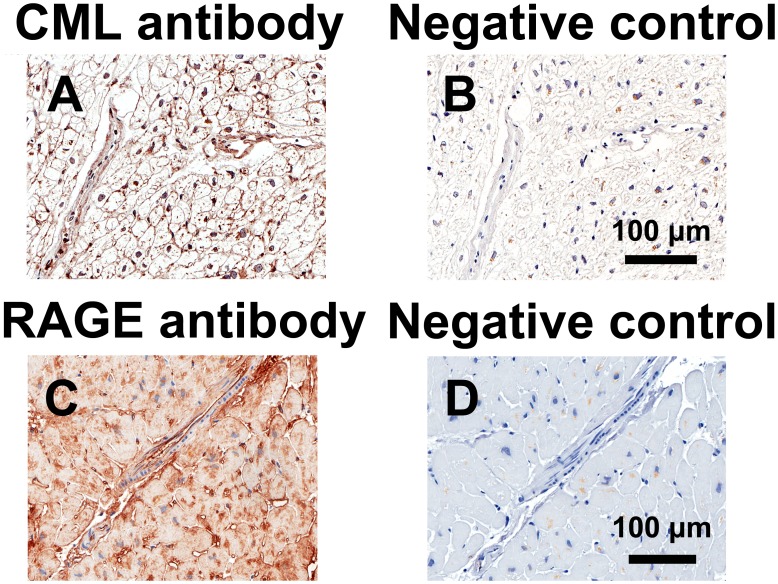
Immunostaining for N^ε^-(carboxymethyl)lysine (CML) and the receptor for advanced glycation end-products (RAGE). Representative sections of left ventricular biopsies from coronary artery bypass graft surgery patients immunostained for CML (A) or RAGE (C) and their corresponding negative control sections (B, D).

**Figure 8:**
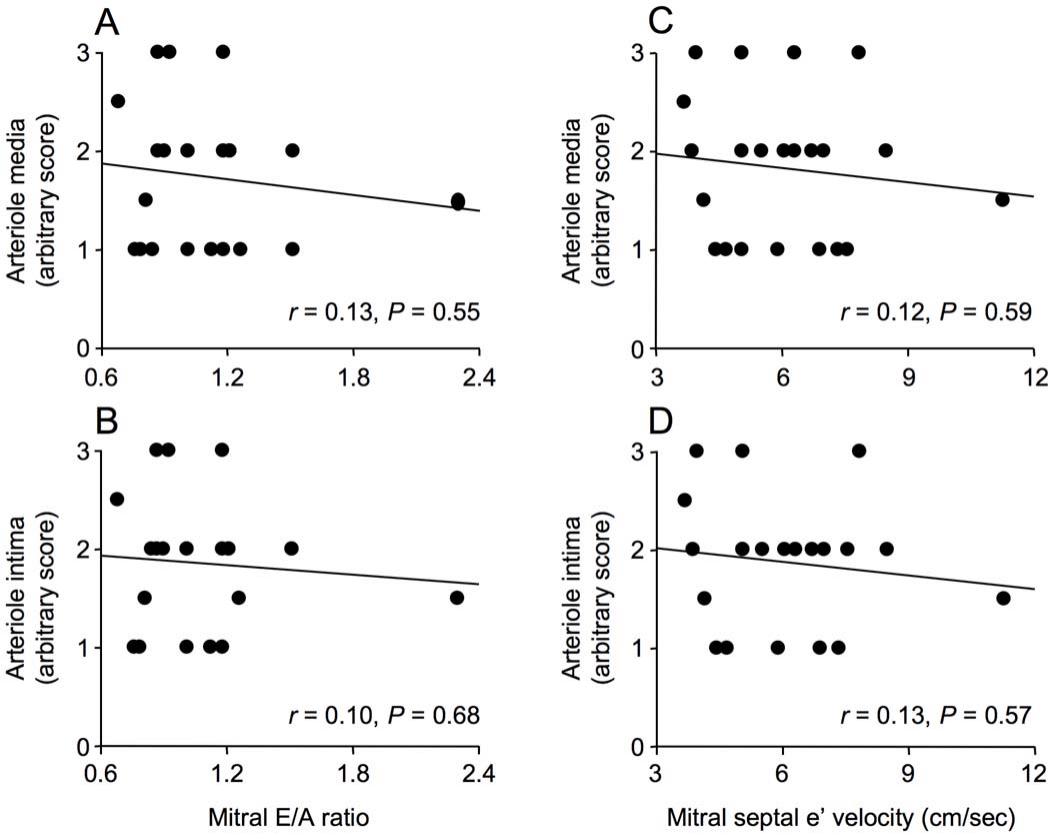
Correlations between N^ε^-(carboxymethyl)lysine (CML) as assessed by immunostaining and echocardiographic parameters of diastolic dysfunction, mitral E/A ratio and mitral septal e ’ **velocity, in men with coronary artery disease.** Immunostaining for CML in arteriolar media and intima was not correlated with mitral E/A ratio (A, B) or with mitral septal e’ velocity (D, E).

**Figure 9:**
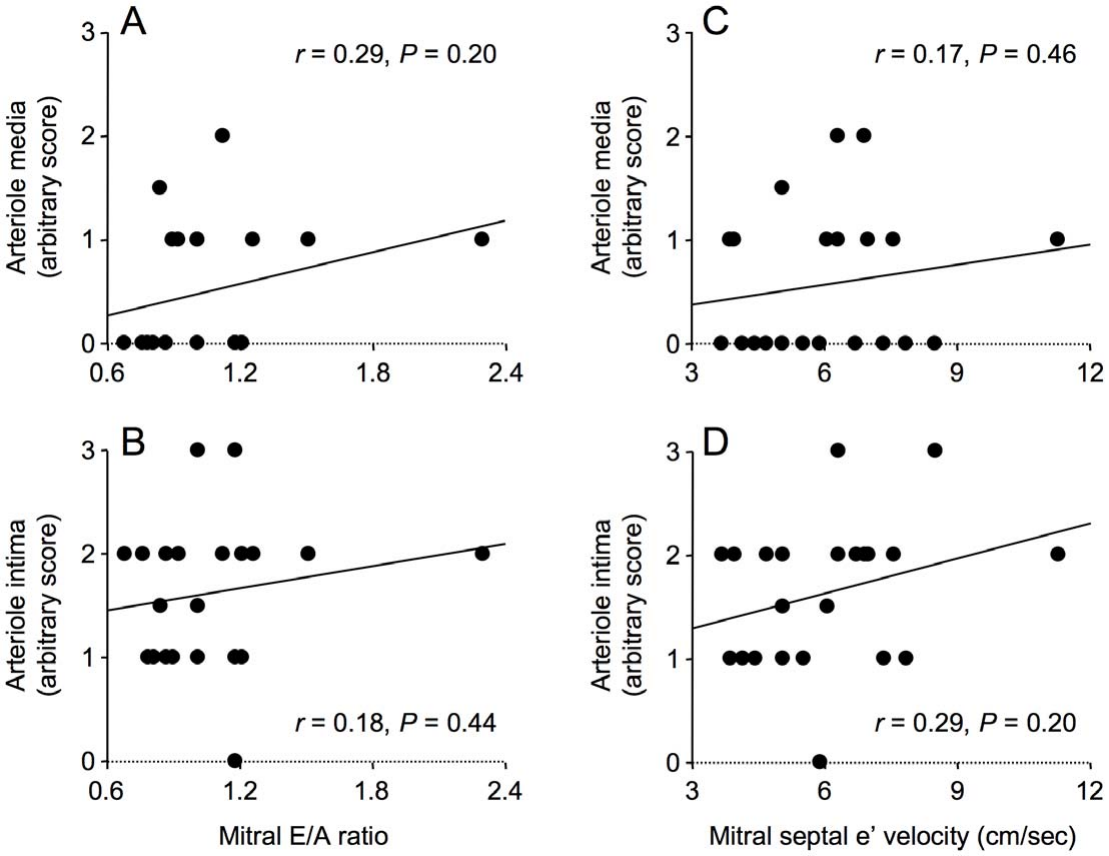
Correlations between the receptor for advanced glycation end-products (RAGE) as assessed by immunostaining and echocardiographic parameters of diastolic dysfunction, mitral E/A ratio and mitral septal e ’ **velocity, in men with coronary artery disease.** Immunostaining for RAGE in arteriolar media and intima was not correlated with mitral E/A ratio (A, B) or with mitral septal e’ velocity (D, E).

Immunostaining for CML was predominantly localized to the media and intima of arterioles and venules, whereas RAGE immunostaining was predominantly localized to the intima of arterioles and venules and to capillaries (Figure 7). Neither CML immunostaining of arteriolar media and intima nor RAGE immunostaining of arteriolar media, intima and capillaries (not shown) was associated with mitral E/A ratio or mitral septal e’ velocity (Figure 8 and 9).

### Plasma Levels of CML, LMWF and Soluble RAGE

**Figure 10:**
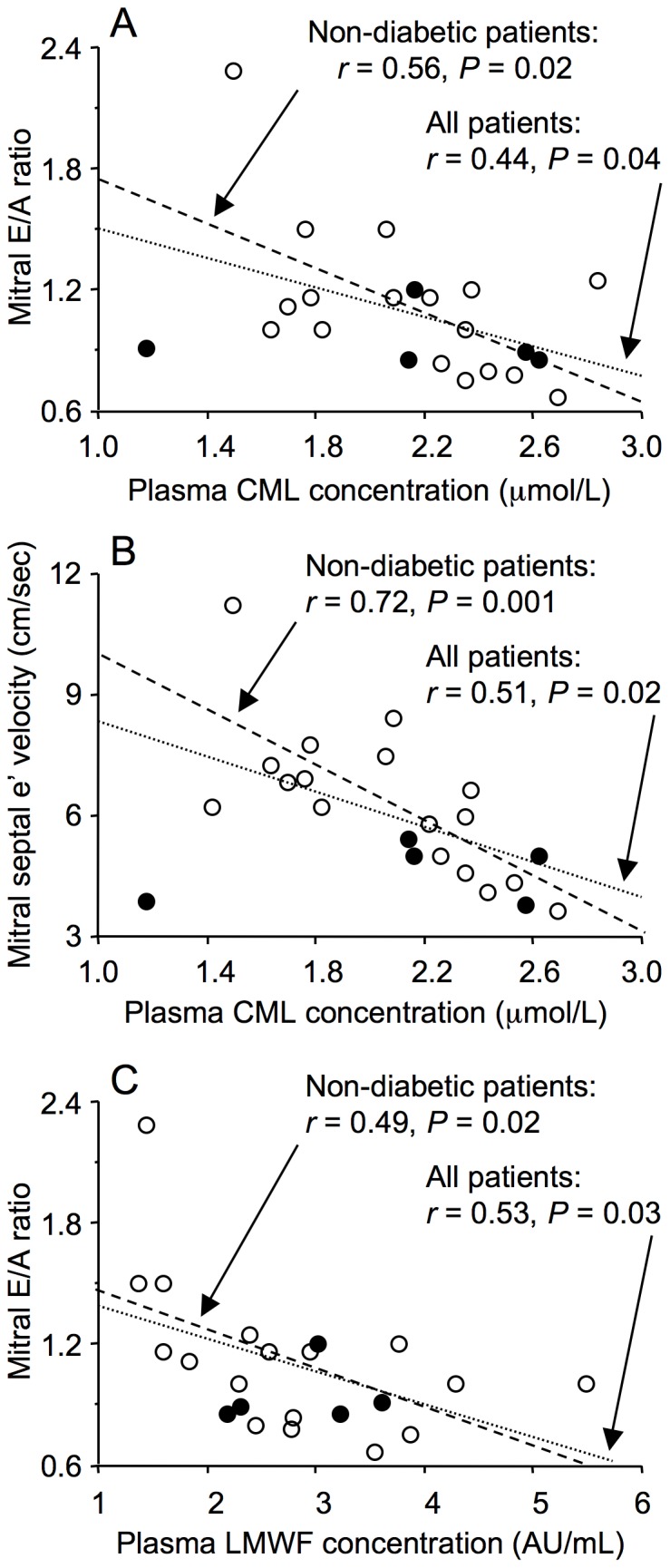
Correlations between plasma advanced glycation end-products and echocardiographic parameters of diastolic dysfunction, mitral E/A ratio and mitral septal e ’ **velocity, in men with coronary artery disease.** Plasma N^ε^-(carboxymethyl)lysine (CML) concentrations were correlated with mitral E/A ratio (A) and mitral septal e’ velocity (B), both for all patients and after exclusion of diabetic patients (non-diabetic patients). In addition, plasma low molecular weight fluorophore (LMWF) concentrations were correlated with mitral E/A ratio (C) for all patients and after exclusion of diabetic patients. Individual values are shown for non-diabetic (open circles) and diabetic patients (closed circles). Correlations are shown for all patients (dotted line) and for non-diabetic patients (dashed line).

Plasma CML levels were not statistically significantly correlated with age in either all patients (*r* = 0.21, *P* = 0.30, n = 26) or non-diabetic patients (*r* = 0.30, *P* = 0.20, n = 20). Similarly, plasma LMWF levels were not statistically significantly correlated with age in either all patients (*r* = 0.25, *P* = 0.22, n = 26) or non-diabetic patients (*r* = 0.28, *P* = 0.23, n = 20). There were, however, significant correlations between plasma levels of CML and LMWFs and echocardiographic indices of diastolic dysfunction (Figure 10). In multivariate analysis with age as a covariate, the standardized coefficients for the correlations between CML, LMWFs and diastolic dysfunction were closer to zero and many were no longer statistically significant, indicating that the association of these AGEs with diastolic dysfunction was dependent in part on their weak, though not statistically significant, correlation with age (Table 2). There was also evidence that the correlation between plasma AGEs and diastolic dysfunction was in part independent of age in that the mitral E/A ratio remained significantly correlated with plasma LMWF levels in all patients, and the mitral septal e’ velocity remained significantly correlated with plasma CML levels in non-diabetic patients when age was a covariate (Table 2). Plasma levels of soluble RAGE did not correlate with any echocardiographic parameter or with eGFR or pulse pressure (not shown).

**Table 2:** Regression analyses between age, plasma N^ε^-(carboxymethyl)lysine (CML) and low molecular weight fluorophore (LMWF) levels, mitral E/A ratio and mitral septal e’ velocity in men with coronary artery disease.

	Variable	All patients, n = 22		Non-diabeticpatients,n = 17	
		Std.coefficient	*P*	Std.coefficient	*P*
Mitral E/A ratio:					
Univariate regression:					
	Age	−0.64	0.001	−0.61	0.010
	CML	−0.44	0.041	−0.56	0.019
	LMWF	−0.49	0.021	−0.53	0.029
Multivariate regression: Age & CML					
	Age	−0.56	0.005	−0.45	0.058
	CML	−0.27	0.14	−0.37	0.11
Multivariate regression: Age & LMWF					
	Age	−0.56	0.003	−0.48	0.023
	LMWF	−0.36	0.036	−0.39	0.065
Mitral septal e’ velocity:					
Univariate regression:					
	Age	−0.55	0.008	−0.55	0.021
	CML	−0.51	0.016	−0.72	0.001
	LMWF	−0.29	0.19	−0.30	0.24
Multivariate regression: Age & CML					
	Age	−0.44	0.026	−0.31	0.12
	CML	−0.38	0.052	−0.59	0.007
Multivariate regression: Age & LMWF					
	Age	−0.51	0.015	−0.51	0.041
	LMWF	−0.18	0.35	−0.17	0.47

St. coefficient, standardized coefficient.

## Discussion

The key finding of this study was that the diastolic dysfunction of aging men undergoing coronary artery bypass graft surgery was not associated with myocardial fibrosis or alteration in cardiomyocyte width, capillary length density, diffusion radius, arteriolar dimensions or myocardial expression of CML and RAGE, but was associated with plasma levels of CML and LMWFs, and these associations were dependent, in part, on age. In addition to diastolic dysfunction, older patients had higher plasma NT-proBNP levels and lower eGFR, consistent with the effects of aging. Our patient population included men with type 2 diabetes and the metabolic syndrome, and in a separate analysis we showed that neither condition affected myocardial structure, microvasculature, or expression of CML and RAGE, apart from reduced perivascular fibrosis of diabetic and metabolic syndrome patients [22]. Although diabetic patients showed evidence of impaired diastolic function [22], the association between plasma AGE levels and diastolic dysfunction in the present study remained after exclusion of diabetic patients. Our findings suggest that the increased myocardial fibrosis, cardiomyocyte hypertrophy and reduced microvascular density of heart failure patients [5]–[9] are a consequence, rather than an initiating cause, of heart failure in the elderly and, in addition, that mechanisms other than alteration in myocardial structure and microvasculature contribute to the diastolic dysfunction of aging.

Echocardiographic indices of diastolic dysfunction are associated with an increased risk of heart failure [40]. Our finding that age was associated with echocardiographic indices of diastolic dysfunction but not with pulmonary capillary wedge pressure was in agreement with previous studies reporting that although associated with alterations with LV filling, age is not associated with invasive measures of pulmonary capillary wedge pressure or with LV isovolumic pressure decay [41]–[43]. Many different mechanisms other than alteration in myocardial structure and microvasculature may contribute to alterations in LV filling and increased heart failure risk of aging individuals [2], [3], [44], including alteration in myocardial energetics. Both myocardial phosphocreatine/adenosine triphosphate ratio and diastolic function are influenced by physical activity status [45], and myocardial phosphocreatine/adenosine triphosphate ratio is reported to be negatively correlated with age [4], [46]. A failure to detect a correlation between age and myocardial phosphocreatine/adenosine triphosphate ratio in other studies may be because of the limited age range of the subjects studied [45], [47], [48]. However, myocardial phosphocreatine/adenosine triphosphate ratio was unrelated to the changes in diastolic function that occur with normal human aging [4], [46]. Other potential mechanisms of diastolic dysfunction of aging include alteration in titin phosphorylation [49], [50], changes in myocardial redox state and impairment of intramyocardial nitric oxide signaling [50], [51].

Previous studies reported plasma CML levels were correlated with the severity and prognosis of heart failure [16] and predicted all-cause and cardiovascular disease mortality in older adults [17]. The present study extends this relationship to a much earlier stage in the evolution of heart failure by demonstrating that plasma CML and LMWF levels correlated with diastolic dysfunction in patients without heart failure. The correlation of plasma, but not myocardial, AGE levels with diastolic dysfunction suggests a role for extra-cardiac AGEs in the diastolic dysfunction of aging, such as may result from increased arterial AGE levels causing reduced arterial compliance [52]. In support of this proposal, Semba et al. [53] found an association between plasma CML levels and aortic pulse wave velocity. The AGE cross-link breaker ALT-711 markedly improves arterial compliance in aged canines [52] and in aged humans with vascular stiffening [54]. However, evidence against a direct relationship between arterial compliance and diastolic function was the failure of ALT-711 therapy-induced improvement in arterial compliance to modify LV diastolic properties [52]. Further studies are required to determine the basis of the relationship between plasma AGE levels and diastolic dysfunction. Our finding of a relationship between plasma AGE levels and diastolic dysfunction in older patients without heart failure suggests that plasma AGE levels may serve as a biomarker for the identification of individuals at increased risk of heart failure.

Myocardial RAGE expression was predominantly localized to vascular endothelium and capillaries, in agreement with previous studies [55]. Whereas cell-surface-bound RAGE plays an essential role in mediating the effects of AGEs, soluble RAGE may act as an AGE inhibitor by sequestering AGEs and preventing their binding to the cell-surface-bound RAGE receptor [56]. Our data suggest that neither myocardial RAGE nor plasma soluble RAGE participate in the diastolic dysfunction of aging.

Our study highlights differences between the effects of aging on the myocardium of humans and animals. In contrast to senescent animals [10]–[13] we found no increase in fibrosis of older patients, in agreement with previous studies in humans [19], [57], and no effect of age on capillary length density and diffusion radius, in agreement with the autopsy study of Roberts and Wearn [18]. Moreover, the lack of effect of age on coronary arteriolar dimensions was in agreement with studies of senescent beagles [11]. Our finding of no effect of age on cardiomyocyte size was in agreement with the autopsy study of Roberts and Wearn [18], but was in contrast to other autopsy studies that showed loss of cardiomyocytes and cardiomyocyte hypertrophy in aging men, but not women [19], [20].

Our study had a number of limitations. The sample size was limited by the need for myocardial biopsies from each patient, with a possibility of type 2 error; our study was therefore largely limited to univariate analyses and many significant correlations may have been missed. In addition, the exclusion of women limited the generalizability of our findings. As expected for patients undergoing coronary artery bypass graft surgery, all had extensive coronary artery disease. However, patients with coronary artery disease were an important group to study because of the high prevalence of coronary artery disease in the community, and there was no association between extent of coronary artery disease and age in our patient population, suggesting that myocardial ischemia was similar across the different ages. Moreover, Redfield et al. [1] found similar age- and gender-related ventricular-vascular stiffening in patients with and without cardiovascular disease. Twelve percent of our patients had wall motion abnormalities but we found no relationship between the presence of wall motion abnormalities and echocardiographic indices of diastolic dysfunction. We took particular care to collect biopsies from non-ischemic myocardium, although we cannot completely exclude the possibility of myocardial ischemia in the biopsied area. However, we found no impact of coronary disease on myocardial structure and microvasculature in a separate study of patients with aortic stenosis with and without coronary disease [23]. Although left ventricular hypertrophy may impact on diastolic function, we found no association between left ventricular mass or cardiomyocyte width and either mitral E/A ratio or mitral septal e’ velocity. The biopsies were from the same subepicardial region of the LV myocardium in all patients and we do not know if the data obtained apply to other regions of the myocardium.

In summary, we showed that the diastolic dysfunction of aging was independent of myocardial structure and microvasculature, but was associated with plasma AGE levels. Our data therefore suggest that the increased myocardial fibrosis, cardiomyocyte hypertrophy and reduced microvascular density of heart failure patients [5]–[9] are a consequence, rather than an initiating cause, of heart failure in the elderly and that mechanisms other than alteration in myocardial structure and microvasculature contribute to the diastolic dysfunction of aging. Although further studies are required to determine the basis of their relationship with diastolic dysfunction, plasma AGE levels may serve as a biomarker to help identify individuals at increased risk of heart failure.
